# Treatment issues in recurrent *Clostridioides difficile* infections and the possible role of germinants

**DOI:** 10.1093/femsmc/xtaa001

**Published:** 2020-09-23

**Authors:** Noah Budi, Nasia Safdar, Warren E Rose

**Affiliations:** School of Pharmacy, University of Wisconsin-Madison, Madison, WI, USA, 53705; Division of Infectious Diseases, University of Wisconsin School of Medicine and Public Health, Madison, WI, USA, 53726; School of Pharmacy, University of Wisconsin-Madison, Madison, WI, USA, 53705

**Keywords:** *difficile*, germination, recurrence, *Clostridioides*, microbiome, colonization resistance

## Abstract

*Clostridioides difficile* is the number one cause of hospital-acquired infections in the United States and one of the CDC's urgent-level pathogen threats. The inflammation caused by pathogenic *C. difficile* results in diarrhea and pseudomembranous colitis. Patients who undergo clinically successful treatment for this disease commonly experience recurrent infections. Current treatment options can eradicate the vegetative cell form of the bacteria but do not impact the spore form, which is impervious to antibiotics and resists conventional environmental cleaning procedures. Antibiotics used in treating *C. difficile* infections (CDI) often do not eradicate the pathogen and can prevent regeneration of the microbiome, leaving them vulnerable to recurrent CDI and future infections upon subsequent non-CDI-directed antibiotic therapy. Addressing the management of *C. difficile* spores in the gastrointestinal (GI) tract is important to make further progress in CDI treatment. Currently, no treatment options focus on reducing GI spores throughout CDI antibiotic therapy. This review focuses on colonization of the GI tract, current treatment options and potential treatment directions emphasizing germinant with antibiotic combinations to prevent recurrent disease.

## Teaser

Schematic of the infectious life cycle of *Clostridioidesdifficile* and treatment opportunity.

## INTRODUCTION

*Clostridioides difficile* is an anaerobic, spore and toxin forming, Gram-positive pathogen that has become the most commonly identified hospital-acquired infection (HAI) in the United States (McDonald *et al*. 2018). *Clostridioides difficile* is one of the CDC's five urgent threat level infections, requiring urgent and aggressive action. Hospital onset infections alone cost an estimated $1 billion in 2017, took 12 800 lives and caused 223 900 infections (CDC 2019). The first step in contracting a *C. difficile* infection (CDI) is ingesting spores through either person-to-person spread via the fecal–oral route, or direct exposure to the contaminated environment (McDonald *et al*. 2018). Once spores reach their ideal intestinal and colonic growth environment, they are introduced to naturally produced chemical signals, referred to as germinants, that convert them into vegetative cells. Vegetative cells then multiply and cause inflammatory disease by producing toxins. Vegetative cells revert into spores that are shed into the environment, increasing HAIs, or remain in the gastrointestinal (GI) tract to be activated later, which is a mechanism for subsequent CDI recurrence. In the treatment of CDI, antibiotics can remove vegetative cells, stopping clinical symptoms, but do not impact spores and can disrupt the normal flora that prevents *C. difficile* growth and CDI recurrence. The GI microbiome plays a protective role in limiting the pathogenicity of this organism, keeping clinical signs and symptoms of CDI at bay, and is often referred to as colonization resistance (CR). Disruption of CR, which results from antibiotic therapy, allows overgrowth of *C. difficile* leading to clinical CDI and increased spore shedding. This review will cover *C. difficile* acquisition and GI tract colonization, limitations and benefits of current treatment options, and potential new treatment directions emphasizing germinant with antibiotic combinations to prevent recurrent disease.

## SPORE SHEDDING

The *C. difficile* spore is the primary vehicle used to transmit infections between patients. Mature spores are heat and ethanol resistant, not affected by antibiotics, and have an unknown lifespan in the environment, making environmental decontamination difficult. Spore shedding occurs in both clinically infected patients and asymptomatic carriers, which act as a reservoir for the pathogen. Gut carriage is estimated to be around 10% in newly admitted hospital patients (Baron *et al*. 2020). Asymptomatic carriage is common after completing CDI-directed antibiotic courses. Therapy results in symptom resolution and undetectable fecal spore levels, but up to 56% of patients will start asymptomatically shedding spores within 4 weeks (Sethi *et al*. 2010). Spores left over in the GI tract or taken up from the environment post-CDI therapy and before CR is re-established are causes of recurrence (Chilton, Pickering and Freeman 2018). High sporulation rates have been tied to recurrent CDI (rCDI), but show strain-to-strain variability and may not be related to strain type, as is typically thought for ribotype 027 epidemic strains (Burns *et al*. 2011; Gómez, Chaves and Orellana 2017; Tijerina *et al*. 2019). rCDI falls into two general categories: (i) relapse, which is a subsequent infection caused by the same strain, likely due to spores left over in the GI tract after antibiotic cessation, and (ii) re-infection, which is caused by a different strain taken up from the environment. Though relapse implies recurrence from spores left over in the GI tract, the same strain can be re-ingested from the patient's environment, making the determination between relapse and re-infection difficult. Likewise, re-infection may be caused from a colonized strain that was not originally detected but present in the patient. In rCDI patients, relapse is the most common cause and affects 52–88% (Chilton, Pickering and Freeman 2018). Differences in molecular techniques used to differentiate rCDI strains and prevalence of epidemic strains at the time of the study impact this proportion (Tang-Feldman *et al*. 2003; Sisto *et al*. 2011; Figueroa *et al*. 2012; Gómez, Chaves and Orellana 2017; Chilton, Pickering and Freeman 2018; Cho *et al*. 2020). The chance of recurrence, either relapse or re-infection, can be as high as 25% for the first, 45% for the second, and reach as high as 65% for subsequent infections (Kelly 2012). Regardless of the origin of spores in rCDI, new methods that limit spore shedding and internal spore levels need to be developed.

Spore shedding is of particular importance in the hospital environment. Hospital equipment, such as blood pressure cuffs, are not easily cleaned with strong bleach like chemicals and detergents often needed to remove spores. Contamination is ubiquitous on bed rails and floors that can be cleaned with such chemicals (Ali, Muzslay and Wilson 2015). Even residing in a room previously occupied by an individual being treated for CDI can be a risk factor for contracting the disease (hazard ratio of 2.35, *P* = 0.01), independent of other well-known CDI risk factors such as age, antibiotic use and proton pump inhibitors (Shaughnessy *et al*. 2011). New methods to decrease spore shedding and limit environmental contamination that leads to HAIs are desperately needed. One method of decontamination has been to introduce germinants into cleaning solutions (Nerandzic and Donskey 2016). Germinants cause spores to convert into vegetative cells that cannot survive in aerobic environments and are easily destroyed by cleaning solutions. One issue with this method is the type of germinants that would be needed to clean a patient's room and ensuring complete cleaning procedures. Germination is most effective when bile acids are combined with divalent cations and amino acids, which would leave behind nutrient residues on hospital surfaces. The authors also noted a delay in methicillin-resistant *Staphylococcus aureus* and vancomycin-resistant enterococci bactericidal activity when germinants were added to quaternary ammonium, though this was short-lived and not observed after 60 min of exposure (Nerandzic and Donskey 2016). Another method being explored is germinant addition to ethanol-based hand sanitizers that resulted in 2 log_10_ CFU reduction in spores after 2 h (Nerandzic and Donskey 2017). This would be a convenient method of spore decontamination, though soap and warm water may be as effective, as already widely available, and it is unclear whether germinants would cause skin irritation (Oughton *et al*. 2009). These are promising environmental cleaning solutions that warrant further investigation. However, it may be more practical to limit the spread of spores into the environment from the source. Converting spores into vegetative cells in the presence of antibiotics through oral administration of germinants before they leave the GI tract may be a valuable approach. Taking advantage of spore biology by increasing germinant signals in the colon could transform spores into cells *in vivo*, which would die upon excretion into aerobic environments or be destroyed by antibiotic presence. This would interrupt the epidemiology of *C. difficile* by diminishing the number of spores shed into the environment that come into contact with other patients. A concern with this method is whether sudden mass germination of internal spores will cause an increase in toxin production leading to further clinical deterioration. This safety concern requires careful investigation in living systems before being used in rCDI patients.

## COLONIZATION RESISTANCE

Antibiotics are a major risk factor for developing CDI as well as rCDI (Garey *et al*. 2008). The reason for this is thought to be from disturbances in the microbiome that remove organisms that outcompete *C. difficile* for resources and changes to the bile acid profile that make a more suitable environment to support vegetative cell growth. Primary bile acids, such as cholate and chenodeoxycholate, can be conjugated with amino acids such as taurine and glycine, secreted into the intestinal tract, and edited by the microbiome into secondary bile acids. Taurocholate, a conjugated primary bile acid, is the strongest known signal for germination and does not inhibit growth of *C. difficile*. Taurocholate can be deconjugated by bile salt hydrolases into cholate, which possesses ∼75% of taurocholate's germination power (Sorg and Sonenshein 2008; Mullish *et al*. 2019). Deoxycholate, the 7α-dehydroxylation product of cholate and a secondary bile acid, can induce germination but inhibits growth of *C. difficile* (Sorg and Sonenshein 2008). Commensal *Clostridia* harboring the *bai* operon are associated with conversion of cholate to deoxycholate and reduced risk of CDI (Solbach *et al*. 2018; Reed *et al*. 2020). Chenodeoxycholate, the other primary bile acid, and its derivatives inhibit both germination and vegetative cell growth (Sorg and Sonenshein 2008). Shifts in the bile acid pool due to antibiotic administration have been evaluated in murine models (Koenigsknecht *et al*. 2015; Theriot, Bowman and Young 2016). After antibiotic administration, taurocholate concentrations increase and anti-germinant and growth-inhibiting bile acids decrease. Taurocholate can reach up to 4 µg per 100 mg of cecal content in antibiotic-treated CDI susceptible mice while almost undetectable in non-antibiotic-treated mice (Koenigsknecht *et al*. 2015; Theriot, Bowman and Young 2016). Similar bile acid changes are seen in the colon and stool (Koenigsknecht *et al*. 2015). This concentration is roughly equivalent to 77.6 nM and is effective at germinating a small population of cecal spores in the absence of competitive inhibitor bile acids *ex vivo* (Theriot, Bowman and Young 2016). However, using Michaelis–Menten kinetics, the concentration of taurocholate to reach half maximal germination rates (Km) is reported at 2–3 mM (Sorg and Sonenshein 2010; Allen *et al*. 2013). The magnitude difference between the Km and taurocholate concentrations found in the cecum, colon and stool indicates a large population of spores is left dormant.

Further research is needed on whether an infection can be prevented, and possibly cured, by administering combinations of these bile acids to patients. Investigation on whether exogenous bile acids allow the natural microbiome to re-establish CR more efficiently would also be beneficial. This differs significantly from current treatments, like vancomycin, which is known to reduce CR (Lewis *et al*. 2015).

## CURRENT CDI TREATMENTS

The Infectious Diseases Society of America and Society for Healthcare Epidemiology of America provide clinical practice guidelines and recommendations for treatment of *C. difficile* (Table 1). Oral vancomycin 125 mg four times daily for 10 days is a recommended treatment and most commonly used for CDI (McDonald *et al*. 2018). Failure to eliminate vegetative cells is unlikely because fecal vancomycin concentrations reach several magnitudes higher than the minimum inhibitory concentration (MIC) for *C. difficile*, similar to the other recommended therapy fidaxomicin (Gonzales *et al*. 2010; Louie *et al*. 2011). In addition, vancomycin fecal concentrations above the MIC can remain elevated for 4–5 days after treatment compared with metronidazole that is only detectable during therapy (Abujamel *et al*. 2013). The main concern with vancomycin is the drastic change to the microbiome that impacts CR and collateral vancomycin resistance to other pathologic organisms that can fill the void (Edlund *et al*. 1997; Louie *et al*. 2009; Lewis *et al*. 2015; Isaac *et al*. 2017).

**Table 1.** Recommendations for the treatment of *C. difficile* infection in adults.*

Clinical definition	Supportive clinical data	Recommended treatment^a^	Strength of recommendation/quality of evidence	Cost^b^
Initial episode, non-severe	Leukocytosis with a white blood cell count of ≤15 000 cells/mL and a serum creatinine level <1.5 mg/dL	VAN 125 mg given four times daily for 10 days, OR	Strong/high	$
		FDX 200 mg given twice daily for 10 days	Strong/high	$$$
		Alternate if above agents are unavailable: metronidazole, 500 mg three times per day by mouth for 10 days	Weak/high	$
Initial episode, severe^c^	Leukocytosis with a white blood cell count of ≥15 000 cells/mL or a serum creatinine level >1.5 mg/dL	VAN, 125 mg four times per day by mouth for 10 days, OR	Strong/high	$
		FDX 200 mg given twice daily for 10 days	Strong/high	$$$
Initial episode, fulminant	Hypotension or shock, ileus, megacolon	VAN, 500 mg four times per day by mouth or by nasogastric tube. If ileus, consider adding rectal instillation of VAN. Intravenously administered metronidazole (500 mg every 8 h) should be administered together with oral or rectal VAN, particularly if ileus is present.	Strong/moderate (oral VAN); weak/low (rectal VAN); strong/moderate (intravenous metronidazole)	$$
First recurrence		VAN 125 mg given four times daily for 10 days if metronidazole was used for the initial episode, OR	Weak/low	$
		Use a prolonged tapered and pulsed VAN regimen if a standard regimen was used for the initial episode (e.g. 125 mg four times per day for 10–14 days, two times per day for a week, once per day for a week and then every 2 or 3 days for 2–8 weeks), OR	Weak/low	$
		FDX 200 mg given twice daily for 10 days if VAN was used for the initial episode	Weak/moderate	$$$
Second or subsequent recurrence		VAN in a tapered and pulsed regimen, OR	Weak/low	$
		VAN, 125 mg four times per day by mouth for 10 days followed by rifaximin 400 mg three times daily for 20 days, OR	Weak/low	$$$
		FDX 200 mg given twice daily for 10 days, OR	Weak/low	$$$
		Fecal microbiota transplantation^d^	Strong/moderate	$$

Abbreviations: FDX, fidaxomicin; VAN, vancomycin.

* This table was adapted with permission from the IDSA guidelines.

a All randomized trials have compared 10-day treatment courses, but some patients (particularly those treated with metronidazole) may have delayed response to treatment and clinicians should consider extending treatment duration to 14 days in those circumstances.

b Costs are based off of AWP for the recommended treatment duration: $ = 0–1000, $$ = 1000–2500, $$$ >2500.

c The criteria proposed for defining severe or fulminant *Clostridioides difficile* infection (CDI) are based on expert opinion. These may need to be reviewed in the future upon publication of prospectively validated severity scores for patients with CDI.

d The opinion of the panel is that appropriate antibiotic treatments for at least two recurrences (i.e. three CDI episodes) should be tried prior to offering fecal microbiota transplantation.

Reducing the residual spore burden is the rationale for tapering and pulsed vancomycin dosing in rCDI. Extending vancomycin exposure time allows for residual spores to germinate and lower concentrations of antibiotic may allow partial return of CR, though results have been mixed and the treatment approach is labeled as a weak recommendation with low-quality evidence in the IDSA guideline (Gentry *et al*. 2017; Sirbu *et al*. 2017; McDonald *et al*. 2018). Additionally, vancomycin has not been shown to reliably reduce spore formation or toxin production in cultures compared with tetracyclines and fidaxomicin and has difficulty eliminating cells in stationary phases of growth, which is when spores and toxins are made (Babakhani *et al*. 2012; Bouillaut *et al*. 2015; Aldape *et al*. 2017). Vancomycin also takes longer to inhibit the outgrowth of newly germinated spores compared with fidaxomicin, which may allow time for toxin production during treatment (Allen *et al*. 2013). The ability of tetracyclines and fidaxomicin to reliably inhibit spore and toxin production and their limited impact on CR are likely why tetracyclines have low association, and can be protective, in developing CDI and why fidaxomicin has better rCDI treatment outcomes (Cornely *et al*. 2012; Tariq *et al*. 2018). However, these drugs, as with all antibiotics, do not impact spores that are already present in the GI tract. Lastly, from a patient's perspective, taking a medication four times a day can be very cumbersome and is inversely associated with medication adherence (Claxton, Cramer and Pierce 2001).

In two phase 3 randomized, double blind trials, oral fidaxomicin 200 mg twice daily for 10 days showed superiority over vancomycin in preventing rCDI as a secondary outcome within 28 days of treatment discontinuation (Cornely *et al*. 2012). The per protocol recurrence rate for fidaxomicin compared with vancomycin in patients without a prior episode was 11.7% vs 22.6% (*P* < 0.001). In patients with a prior episode, fidaxomicin saw 19.7% of patients experience recurrence compared with 35.5% for vancomycin (*P* = 0.045). Though relapse rates differed, the primary outcome of clinical cure as assessed by resolution of diarrhea at the end of 10 days of treatment was similar between the two groups (Crook *et al*. 2012). Interestingly, this reduction in rCDI may be strain specific, as rCDI rates do not differ when treating the epidemic NAP1/BI/027 strain (Louie *et al*. 2011). Fidaxomicin's lower recurrence rate is likely attributable to its narrower spectrum of activity compared with vancomycin, preserving aspects of CR (Louie *et al*. 2012). Fidaxomicin also has the ability to associate with spores, persisting on the surface through hydrophobic interactions that may last until the spore germinates, keeping the antibiotic in close proximity (Chilton *et al*. 2016). Vancomycin does not adhere to spores in the same manner as fidaxomicin (Chilton *et al*. 2016). In addition, decreases in spore and toxin production associated with fidaxomicin,  an inhibitor of bacterial RNA polymerase, may contribute to its increased efficacy.

As *C. difficile* toxins A and B are a significant contributor to disease severity, anti-toxin therapeutics have been developed. A single dose of bezlotoxumab 10 mg/kg IV, a monoclonal antibody directed against toxin B, after a CDI-directed antibiotic course has shown similar efficacy in reducing rCDI to fidaxomicin. In two phase 3 randomized trials, the combined recurrence rate for bezlotoxumab was 17% compared with 27% for standard of care (Wilcox *et al*. 2017). Unfortunately, this study was not designed to determine whether the combination of fidaxomicin with bezlotoxumab could further decrease rCDI, and this approach would be cost prohibitive in its broad application to patients. The development of an antibody toward toxin A, actoxumab, was halted due to its lack of effect against rCDI (Wilcox *et al*. 2017). It is unknown why using an antibody directed toward toxin B reduces recurrence, while toxin A is ineffective. It could be related to the potency of the toxins, with toxin B possessing 100–1000× the toxicity of A *in vitro*, and inhibition of this toxin alone may be enough to diminish clinical symptoms (Chaves-Olarte *et al*. 1997). Further information on how toxin B sequestration effects the life cycle of *C. difficile* is needed. Information on spore shedding in this population would also be beneficial pertaining to contact precautions and prevention of HAIs.

Fecal microbiota transplantation (FMT), the instillation of healthy donor feces into a patient's GI tract after CDI directed antibiotic therapy, is recommended for patients with multiple rCDI episodes who have failed appropriate antibiotic treatment. Restoring CR with FMT helps reverse the damage caused by multiple rounds of antibiotic treatment, removing the niche that *C. difficile* needs to grow. Efficacy for FMT varies, but is close to 90% in patients who have had multiple rCDI episodes, the highest risk population for rCDI (Mattila *et al*. 2012). The high success rate of this procedure likely involves changing the bile acid profile, in conjunction with resource competition, as bacteria in FMT possessing bile salt hydrolases or the *bai* operon have been tied to efficacy (Solbach *et al*. 2018; Mullish *et al*. 2019). Changing the bile acid profile from a taurocholate rich environment to one of anti-germinating and growth inhibiting bile acids likely prevents the level of growth needed to cause clinical disease. Information on spore shedding post-FMT is limited, but likely to occur as asymptomatic carriage post-antibiotic treatment for CDI is common (Sethi *et al*. 2010). Antibiotic administration after FMT has resulted in CDI, suggesting patients were not decolonized or that spores persisted in their environment until ingested during subsequent dysbiosis (Mattila *et al*. 2012; Allegretti *et al*. 2019). A major concern with FMT is transmitting infectious agents between patients. One example includes extended spectrum beta-lactamase *E. coli* bacteremia that resulted in the death of a patient (DeFilipp *et al*. 2019). Developing the framework within a hospital system to screen donor stool for transplantation can be another barrier. However, this has been partially alleviated through commercially available pre-screened and ready-to-use preparations.

Another promising non-antibiotic treatment being explored, similar to FMT restoration of CR, is oral administration of non-toxigenic *C. difficile* (NTCD) spores post-CDI-directed antibiotic therapy. In a phase 2, randomized, double blind, placebo-controlled trial, patients were given differing doses of NTCD strain M3 spores for 7 or 14 days (Gerding *et al*. 2015). Patients were first treated with courses of vancomycin or metronidazole, two of the CDI-directed antibiotics with the highest recurrence rates, and given NTCD spores or placebo starting 2 days after completion of antibiotics. Patients given placebo experienced higher rates of rCDI at 30% compared with 11% of patients in the NTCD group (OR 0.28 95%, CI 0.11–0.68, *P* = 0.006). The adverse effect profile of spore administration was minimal, and often less than that of placebo. It should be noted that the majority of patients treated were not rCDI patients and multiple rCDI patients were excluded from participation. Lastly, this form of CR, which is likely due to resource competition, may be particularly cost effective to produce through culture. The same durability that allows spores to survive in the environment should afford a long shelf life for hospital pharmacies. The results of this trial are encouraging though more information is needed, particularly in multiple rCDI patients.

## OPPORTUNITIES FOR NEW TREATMENT APPROACHES

**Figure 1.**
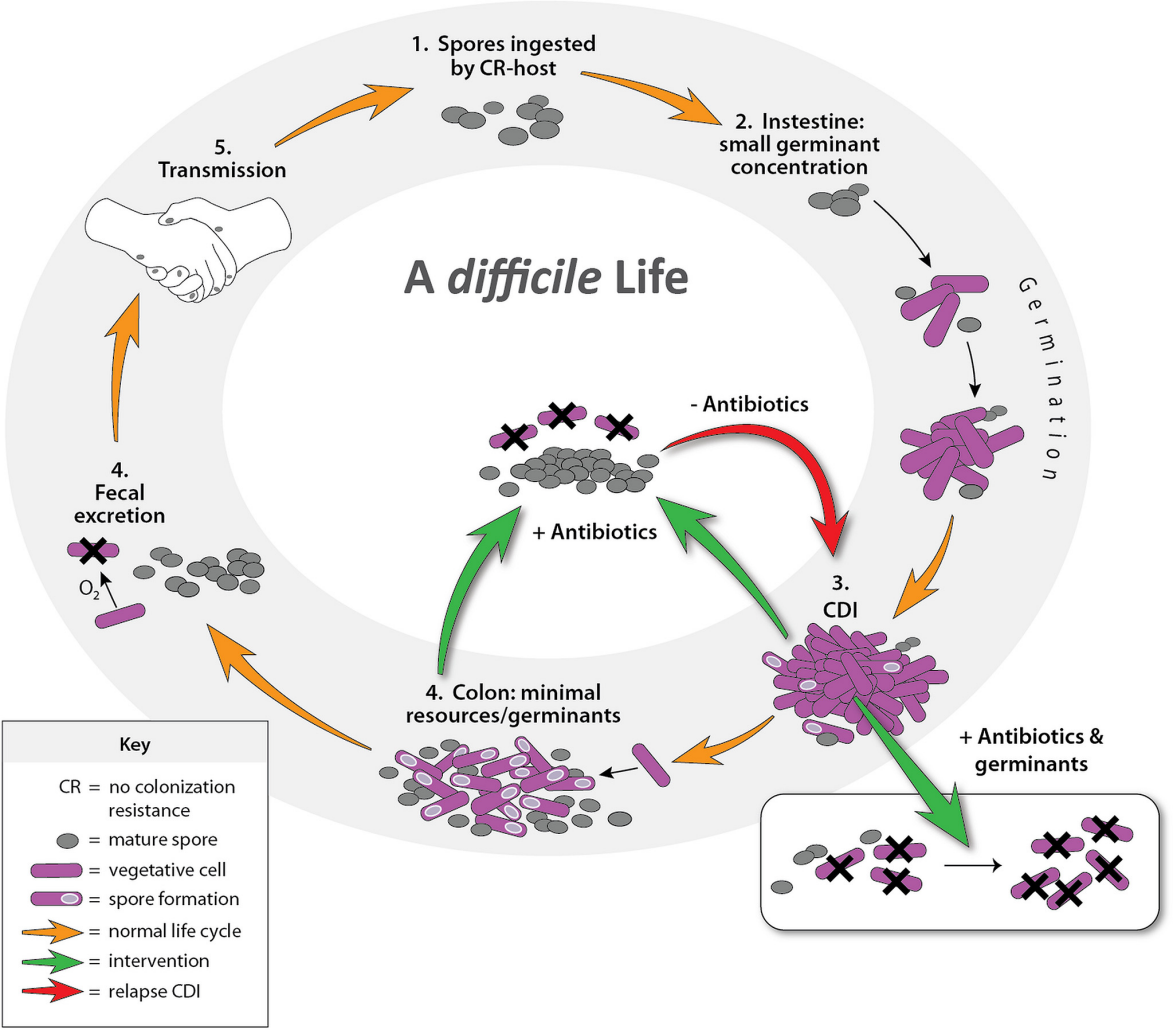
Schematic of the infectious life cycle of *C. difficile* and treatment opportunities.

Spore shedding and persistence in the GI tract are the greatest barriers in preventing rCDI. This was partially elucidated in an murine model with genetically modified strains of *C. difficile* that are unable to produce spores (Deakin *et al*. 2012). Spore negative strains do not cause recurrence, yet colonizing other mice with wild-type, spore-producing strains did. Hypotheses that increased germination rates may be responsible for more severe disease have been refuted (Carlson *et al*. 2015). More stringent control over germination likely allows spores to accumulate during both asymptomatic carriage and active infections. These germination resistant spores may outlast CDI antibiotic therapy and re-establish infection before CR returns. Spores that readily germinate would turn into viable cells during the treatment process and be eliminated by antibiotics. Likewise, if spores are too resistant to germination, they may not be able to outpace regeneration of CR post-CDI antibiotic therapy. This creates an optimal window for germination and outgrowth, after antibiotics wash out from the GI tract following treatment and before CR is replenished. In the case of vancomycin treatment, this optimal window would be between day 5 and 21 after discontinuing therapy in many patients, though the 21-day window may be extended in select patients (Abujamel *et al*. 2013). Replenishing CR with FMT removes this window, but may put patients at risk for CDI during future antibiotic treatment, as spores are likely still present. A solution to these problems might be found in using supraphysiologic germinant concentrations to decolonize patients. Figure 1 is a conceptual illustration of the germinant-antibiotic approach to disrupt the *C. difficile* spore-cell life cycle.

Administering germinants to patients during CDI antibiotic therapy may sufficiently diminish the spore burden to allow CR to regenerate. This approach would leave behind only hyperdormant spores, decrease total antibiotic administration time and may provide a decolonization method for rCDI patients. Additionally, decreased spore shedding in the hospital environment would likely lead to reductions in HAIs. The primary concern with this method is a sudden increase in toxin production and subsequent decline in clinical status. However, understanding that certain antibiotics, such as fidaxomicin and tetracyclines, decrease toxin production, and bezlotoxumab binds toxin B to neutralize its toxigenic effect should mitigate these concerns. In addition to inducing germination, taurocholate, at concentrations as low as 5 mM, has been found to mitigate cytotoxic effects of toxins A and B *ex vivo* (Darkoh *et al*. 2013). Decreased activity occurred without changing toxin concentration, although the mechanism remains unclear (Darkoh *et al*. 2013). Administering germinants could also be timed effectively, allowing for vegetative cells to be removed with antibiotics before germinating spores, limiting the active bacterial load. Toxin production occurs during stationary phases of growth, when vegetative cell burden is high, and may be related to quorum sensing (Darkoh *et al*. 2015). Careful investigation is needed to determine whether newly germinated spores at levels found within the host are able to produce toxins in antibiotic presence.

Additional considerations are the types of germinants that would be needed for transition from spore to vegetative cell. Taurocholate is taken up in the small intestine and recycled in the bile acid pool. Creation of a non-absorbable version of taurocholate would be beneficial to assure that the dose is reaching the colon in significant amounts. Alternatively, inhibitors of the apical sodium-dependent bile acid transporter, responsible for bile acid uptake, have recently been developed to aid insulin responses in type 2 diabetes and prevent itching in primary biliary cholangitis (Hegade *et al*. 2017; Al-Dury and Marschall 2018). Oral administration of taurocholate has been tested for other indications and was well tolerated when given at doses as high as 1 g every 8 h over 2 days, though the number of patients tested was small (Plusa and Clark 1991). Calcium, a strong co-germinant, is only 25% bioavailable when given as calcium carbonate, and should not have issue reaching significant concentrations to induce germination (Hunt and Johnson 2007). The specific co-germinants required vary between strains, but usually include a divalent cation and amino acids, both of which should be safe to administer to patients (Shrestha and Sorg 2018).

The final consideration in using germination to eradicate spores is hyperdormancy, or spores that do not readily vegetate in response to germinants. This may be due to the spore's outermost layer, the exosporium. The bile acid receptor CspC implicated in initiating germination is hypothesized to be in the spore coat or outer membrane, both located under the exosporium (Kochan *et al*. 2018). In addition, exosporium removal increases the spore's ability to form colonies (Calderón *et al*. 2018). The exosporium aids in chemical and heat resistance and increases hydrophobicity, which may allow it to adhere to environmental surfaces and colonic mucosa (Paredes-Sabja and Sarker 2012; Calderón *et al*. 2018). This layer also degrades over time, which may aid in preventing germination until antibiotics are washed out of the GI tract (Pizarro-Guajardo, Calderón-Romero and Paredes-Sabja 2016). This raises concerns, as previous experiments looking at germination efficiency often include multiple washing steps before the introduction of germinants. Unfortunately, it would not be possible to remove this layer *in vivo* with conventional methods such as sonication, centrifugation or enzyme digestion. However, hyperdormant spores may not germinate in sufficient numbers or in a timely manner to outpace the regeneration of CR.

## CONCLUSION

Spore shedding and colonization of the GI tract are significant barriers for preventing HAIs and treating rCDI patients. Options to remove toxigenic spores from a patient's GI tract are severely limited and progressive CDI antibiotic usage may prevent re-establishment of CR. FMT has been very successful at preventing rCDI, but its efficacy is removed through subsequent antibiotic treatment. Currently, no therapy exists to address this issue. Turning spores into vegetative cells with germinants for antibiotic targeting is an alternative approach with potential to decrease the prevalence of CDI. Future studies should investigate the effect and safety of germinants with antibiotics in animal models of CDI to determine whether combination therapy has potential benefit.

## Supplementary Material

xtaa001_Supplemental_File
