# Supplementary material for: Treatment issues in recurrent Clostridioides difficile infections and the possible role of germinants
Source: FEMS Microbes. 2020 Sep 23;1(1):xtaa001. doi: 10.1093/femsmc/xtaa001 (PMC10117431; doi:10.1093/femsmc/xtaa001)
Supplement: xtaa001_Supplemental_File [file xtaa001_supplemental_file.docx]

**Manuscript Title: Treatment Issues in Recurrent Clostridioides difficile Infections and the Possible Role of Germinants**

**Authors: Budi, Noah; Safdar, Nasia; Rose, Warren**

**Manuscript ID: FEMSMC-2020-001.R1**

**Reviewers' Comments to Authors on Original Manuscript:**

**Reviewer: 1**

Comments to the Author


Title: Disrupting the Life Cycle of Clostridioides difficile to Improve Antibiotic Treatment

General: this is a general review focused on multiple aspects of CDI.  The paper covers the life cycle of CDI, some aspects of spore germination, current IDSA treatment recommendations, a brief review of the microbiome, colonization resistance, and fecal microbiota transplantation, and then finished with emerging therapies somewhat focusing on potential role of germinants as a new treatment modality.  This is very ambitious to say the least.  I have several major and minor comments

Major comments:
1. The review needs to be significantly streamlined.  It seems that the authors are most interested in better understanding spore germination and the potential therapeutic potential of interfering with this life cycle.  This could be a fascinating review with plenty of potential.  If I am correct in this assumption, the authors need to significantly streamline this review
2. The authors need to provide a science-based perspective on their review.  It seems to me that the authors are advocating using treatments to induce germination in the gut.  This seems like a preliminary and potentially dangerous recommendation.  Any potential recommendations needs to be based on scientific fact
3. Search strategy:  A Prisma flow chart, defined search strategy, and more precise objectives would significantly improve this manuscript.

Minor comments:
• Title doesn’t reflect the actual content of the paper
• Abstract draws inaccurate information about the topic
• Extensive proofreading is needed to minimize typos, spelling, and grammars mistakes
• The transition between ideas need improvement and rephrasing
• Complete inappropriate referencing such as reference 2 and 55…etc.
• Inappropriate and/or unclear citations
• Page 5 Line 47-52: conflicting information about bile acid and germination
• Need to define some abbreviations like MRSA and VRE
• Page 3 Line 54: causation needs more supportive evidence
• Page 4 Line 19: too much references for one sentence
• Page 13 Line 23: the suggested approach (as novel) could be worded as alternative strategy for rCDI
• Several places: very long sentences break the meaning and makes it unclear
• Page 12 Line 44-45: even evidence from your lab work needed to be published and cited
• Change wording to more scientific terms for example:
o Spore shedding and persistence in GI= colonization
o Patients with asymptomatic carriage= asymptomatic carriers
o Removal of CR= disruption in CR
o Spores removal= eradication

Tables and figure:
• Table1: well-constructed, however the two tables are the same in their content with different titles.  Is this an exact replica of the IDSA guidelines?
• Figure1: very well explained and constructed.


**Reviewer: 2**

Comments to the Author
Title: Disrupting the life cycle of Clostridioides difficile to improve antibiotic treatment

In this manuscript, Budi and colleagues provide an in-depth overview of C. difficile infection.  Specifically, the authors focus on the role for the infectious C. difficile spore in disease manifestation, persistence, and recurrence.  In addition to a comprehensive review on the basic and clinical significance of the C. difficile spore, the authors provide insights into novel strategies to target the spore and improve treatment of C. difficile infection. Overall, this review is well written, informative, and provides interesting insights into an important, yet underappreciated area of the C. difficile infectious cycle.  Below are specific comments for the authors to address.


1. The authors should comment on sporulation rates in various strains of C. difficile.  Is this associated with increased rates of infection, recurrence, etc.?
2. How do sporulation deficient strains of C. difficile alter disease outcomes and relapse rates in animal models?
3. The section detailing colonization resistance should be expanded on.  More information on the role of primary and secondary bile acids would be helpful.  Moreover, the authors should provide more detail on specific roles for members of the microbiota in manipulations of the germinant pool (e.g. bile acids).  This could include recent findings describing antagonistic Clostridia and the bai operon.
4. In the section detailing new therapeutics and the potential use of germinants for treatment of C. difficile infection, the authors should describe the potential impact of these treatments may have on other members of the microbiota. What is known about the impact of bile acids on other commensals?
5. What are the levels of primary bile acids during the course of C. difficile infection?  My understanding is that primary bile acids, like taurocholate, are enriched in the stools of patients during infection and following vancomycin treatment.  Despite this, spores continue to persist in the colon, as detailed in this review.  How would introduction of more germinant change this?  
6. The authors should include more information on the mechanisms and role of sporulation in C. difficile.  In their section on new treatment approaches, discussing the potential of targeting and limiting sporulation, would be an interesting addition to directly targeting the spore.

**FEMSMC-2020-001**

**Authors’ Response**

| **Author's Response to Decision Letter for (FEMSMC-2020-001)** |
| --- |
| **Disrupting the Life Cycle of *Clostridioides difficile* to Improve Antibiotic Treatment** |
| We thank the reviewers and editor for taking the time to evaluate our manuscript. The peer review process is a vital part of scientific literature and was extremely helpful in allowing us to clarify concepts for your readers. We are particularly thankful for the notification on incomplete and inappropriate citations. This appeared to happen in the submission process and is unacceptable.  Reviewer concerns have been addressed below. In addition, a track changes version has also been uploaded along with a revised version.  Responses to reviewer comments for: “Treatment Issues in Recurrent Clostridioides difficile Infections and the Possible Role of Germinants”  Reviewer: 1 Comments to the Author  Title: Disrupting the Life Cycle of Clostridioides difficile to Improve Antibiotic Treatment  General: this is a general review focused on multiple aspects of CDI. The paper covers the life cycle of CDI, some aspects of spore germination, current IDSA treatment recommendations, a brief review of the microbiome, colonization resistance, and fecal microbiota transplantation, and then finished with emerging therapies somewhat focusing on potential role of germinants as a new treatment modality. This is very ambitious to say the least. I have several major and minor comments  Major comments:  The review needs to be significantly streamlined. It seems that the authors are most interested in better understanding spore germination and the potential therapeutic potential of interfering with this life cycle. This could be a fascinating review with plenty of potential. If I am correct in this assumption, the authors need to significantly streamline this review. Response: Streamlining of the review was balanced with the other reviewer’s requests for more information on the topic. When possible, sentences were condensed and redundant examples were removed.    The authors need to provide a science-based perspective on their review. It seems to me that the authors are advocating using treatments to induce germination in the gut. This seems like a preliminary and potentially dangerous recommendation. Any potential recommendations needs to be based on scientific fact. Response: This paper is designed to review the CDI recurrence problem and hypothesizes new approaches on how to address this issue. We appreciate this comment from the reviewer, and we have improved science-based perspective for forming this hypothesis. Throughout the paper, we have now referenced fully the issue of residual spores and recurrent CDI following antibiotic therapy alone. We have reinforced that germinant/antibiotic combinations need further investigation for safety before implementation in rCDI patients can be considered. Information on when toxin production occurs and evidence that germinant addition to in vitro cultures does not lead to increased toxin production have been included with references.    Search strategy: A Prisma flow chart, defined search strategy, and more precise objectives would significantly improve this manuscript. Response: This review captures multiple aspects of CDI and the number of search terms that would have to be incorporated would make a flow chart or defined search strategy difficult. The topics covered are intimately related to one another and discussing one without the others could dilute their individual importance. However, the stated focusses of the review have been edited to more precisely reflect streamlined content.  Minor comments:  Title doesn’t reflect the actual content of the paper Response: The title has been changed from “Disrupting the Life Cycle of Clostridioides difficile to Improve Antibiotic Treatment” to “Treatment Issues in Recurrent Clostridioides difficile Infections and the Possible Role of Germinants”    Abstract draws inaccurate information about the topic Response: It is unclear what information was inaccurate in the abstract as multiple citations in the body of the paper reinforce the information. However, the focusses stated in the last sentence have been rephrased to reflect streamlining of content.    Extensive proofreading is needed to minimize typos, spelling, and grammars mistakes Response: The review has been proofread by the authors for typos, spelling mistakes, and grammatical errors.    The transition between ideas need improvement and rephrasing Response: The transition statements have been edited to enhance the flow of the review.    Complete inappropriate referencing such as reference 2 and 55…etc. Response: The authors are very thankful for this observation. The citation manager autocorrected citations to inappropriate forms. This has been addressed within the citation manager and all citations should now follow AMA formatting. It should be noted that track changes function does not capture changes to citations within the references section.    Inappropriate and/or unclear citations Response: Citations have been double checked for discrepancies and addressed.    Page 5 Line 47-52: conflicting information about bile acid and germination Response: This section has been re-written for clarification. It now starts with descriptions of bile acids, how they interact with C. difficile, and how concentrations are changed during antibiotic administration in murine models.    Need to define some abbreviations like MRSA and VRE Response: MRSA and VRE are now spelled out in the paper and we have checked for other abbreviations that require clarification.    Page 3 Line 54: causation needs more supportive evidence Response: We have improved the supportive evidence for causation. Evidence for colonization resistance preventing CDI can be found in the colonization resistance section. We changed wording from “are the causes of recurrence” to “are causes of recurrence” and added a citation that describes the difference between relapse and reinfection. Additionally, the sentences following th statement describe the issues with determining relapse from reinfection.    Page 4 Line 19: too much references for one sentence Response: Three of the references have been combined into one that reviews them. The remaining references highlight the difficulty in determining relapse from re-infection and how new molecular techniques and emergence of epidemic strains has changed this proportion.    Page 13 Line 23: the suggested approach (as novel) could be worded as alternative strategy for rCDI Response: We have changed this to an alternative approach strategy for rCDI, which aligns with further statements suggesting research required around this approach for clinical translation.    Several places: very long sentences break the meaning and makes it unclear Response: Longer sentences have been condensed and clarified.    Page 12 Line 44-45: even evidence from your lab work needed to be published and cited Response: The reference to our lab has been replaced with information on germinant receptor location.    Change wording to more scientific terms for example: Spore shedding and persistence in GI= colonization Patients with asymptomatic carriage= asymptomatic carriers Removal of CR= disruption in CR Spores removal= eradication Response: All wording changes were done as recommended.  Tables and figure:  Table1: well-constructed, however the two tables are the same in their content with different titles. Is this an exact replica of the IDSA guidelines? Response: Yes, this was adapted with permission and with the addition of cost based on AWP. Please see * in the title. The title is the same with the addition of “guideline” and changing “Clostridium” to “Clostridioides.” The authors determined the inclusion of this table would be helpful for readers to refer to during the treatments section.  Figure1: very well explained and constructed. Response: Thank you.    Reviewer: 2 Comments to the Author   Title: Disrupting the life cycle of Clostridioides difficile to improve antibiotic treatment  In this manuscript, Budi and colleagues provide an in-depth overview of C. difficile infection. Specifically, the authors focus on the role for the infectious C. difficile spore in disease manifestation, persistence, and recurrence. In addition to a comprehensive review on the basic and clinical significance of the C. difficile spore, the authors povide insights into novel strategies to target the spore and improve treatment of C. difficile infection. Overall, this review is well written, informative, and provides interesting insights into an important, yet underappreciated area of the C. difficile infectious cycle. Below are specific comments for the authors to address.   The authors should comment on sporulation rates in various strains of C. difficile. Is this associated with increased rates of infection, recurrence, etc.? Response: The associations between high sporulation rates and rCDI are now discussed in the spore shedding section.    How do sporulation deficient strains of C. difficile alter disease outcomes and relapse rates in animal models? Response: The study “The Clostridium difficile spo0A Gene Is a Persistence and Transmission Factor” has been included in the opportunities section. This study shows spores must be present for C. difficile to effectively transmit between mice and cause recurrence. The study also states that environmental contamination is responsible for recurrence because the authors tried, and failed, to culture spores from intestinal tracts after five days of vancomycin treatment. However, they did not wash fecal samples before plating and vancomycin can take several days to wash out of the system. Methods section does not state if they washed intestinal contents prior to plating. The authors were not available for comment.    The section detailing colonization resistance should be expanded on. More information on the role of primary and secondary bile acids would be helpful. Moreover, the authors should provide more detail on specific roles for members of the microbiota in manipulations of the germinant pool (e.g. bile acids). This could include recent findings describing antagonistic Clostridia and the bai operon. Response: The study “Strain-Dependent Inhibition of Clostridioides difficile by Commensal Clostridia Carrying the Bile Acid-Inducible (bai) Operon” has been included in the colonization resistance section. The section on bile acids has been re-written for clarity. An in-depth analysis of how different members of the microbiome conribute was not included to balance the other reviewer’s request for streamlining.    In the section detailing new therapeutics and the potential use of germinants for treatment of C. difficile infection, the authors should describe the potential impact of these treatments may have on other members of the microbiota. What is known about the impact of bile acids on other commensals? Response: Endogenous bile acids have an antimicrobial effect that regulates the composition of the microbiota as noted in the colonization resistance section. However, information on how taurocholate specifically interacts with this community, especially one experiencing dysbiosis seen in CDI, is unavailable and was not added.    What are the levels of primary bile acids during the course of C. difficile infection? My understanding is that primary bile acids, like taurocholate, are enriched in the stools of patients during infection and following vancomycin treatment. Despite this, spores continue to persist in the colon, as detailed in this review. How would introduction of more germinant change this? Response: The level of taurocholate in comparison to other inhibitory bile acids in the cecum, colon, and stool has been elaborated on in the colonization resistance section. The amount of taurocholate required for half maximal germination rates is compared to the concentrations found after antibiotic administration.    The authors should include more information on the mechanisms and role of sporulation in C. difficile. In their section on new treatment approaches, discussing the potential of targeting and limiting sporulation, would be an interesting addition to directly targeting the spore. Response: The ability of fidaxomicin and tetracyclines to limit spore production is briefly compared to vancomycin in the current treatments section. However, we dcided to focus on the spore burden left after treatment with antibiotics. Because spores and toxins are created at similar times, patients presenting with clinical CDI would already have a spore reservoir. Inhibiting sporulation would be an interesting addition, particularly as it pertains to CDI prevention, but was ultimately not included per the other reviewers request for streamlining. |

**FEMSMC-2020-001.R1**

**Reviewers' Comments to Authors on Revised Manuscript:**

**Reviewer 1**

The authors have addressed my comments adequately. The scope is still somewhat broad but provides a good review of CDI with a specific focus on the role of germinants. No further comments.

**Reviewer 2**

Title: Treatment Issues in Recurrent Clostridioides difficile Infections and the Possible Role of Germinants

In this revised review article, Budi and colleagues provide an in-depth overview of C. difficile infection with a focus on C. difficile spores. The authors have addressed the majority of my concerns and comments, and this review is well written, informative, and provides interesting insights into an important area of the C. difficile infection. Below are some minor comments:

1.The model figure (Figure 1) is busy and somewhat confusing to digest. One suggestion for clarity is breaking the model into 3 panels instead of one. Panel 1.) model with the normal infectious cycle, Panel 2.) for antibiotic treatment and relapsing infection, and Panel 3.) for novel treatment options. Breaking the figure out this way will make it easier to digest where treatment failure occurs and where new opportunities may be leveraged.
